# Supplementary material for: Examining charitable giving in real-world online donations
Source: Nat Commun. 2019 Sep 3;10:3968. doi: 10.1038/s41467-019-11852-z (PMC6722054; doi:10.1038/s41467-019-11852-z)
Supplement: Supplementary file 1 — Supplementary Information [file 41467_2019_11852_MOESM1_ESM.pdf]

Supplementary Information

for the paper: "Examining charitable giving in real-world online donations"

Matthew R. Sisco and Elke U. Weber

Contents

1 Supplementary Note 1 **2**

2 Supplementary Methods **4**

2.1 Gender estimation . . . 4
2.2 Descriptive statistics . . . 6
2.3 Female and male donations . . . 10
2.4 Regressions . . . 13
2.5 Empathy analysis . . . 25
2.6 Simulation: same name by chance . . . 29
2.7 Simulation: controlling for campaign effects . . . 31
2.8 Robustness check: investigating time as a confound . . . 35
2.9 Sensitivity (Post hoc) Power Analysis . . . 38
2.10 Survey data analysis . . . 39
2.11 Survey instrument . . . 42

1 Supplementary Note 1

This supplementary information document contains all of the supplementary figures, tables, and methdods referenced in the paper: *Examining charitable giving in real-world online donations.* Combined with the files "Source_data_file_A.csv" and "Source_data_file_B.csv" this document supplies all code and data needed to exactly reproduce every analysis reported in the paper. We also include various additional analyses and figures to provide further information about the results.

All code in this document was executed in the programming language R version 3.5.1 (and displayed here in the grey boxes). Output from executed code is displayed in black text below the code snippets where it is informative to show.

If you have any questions about the contents of this document please contact Matthew Sisco, ms4403@columbia.edu.

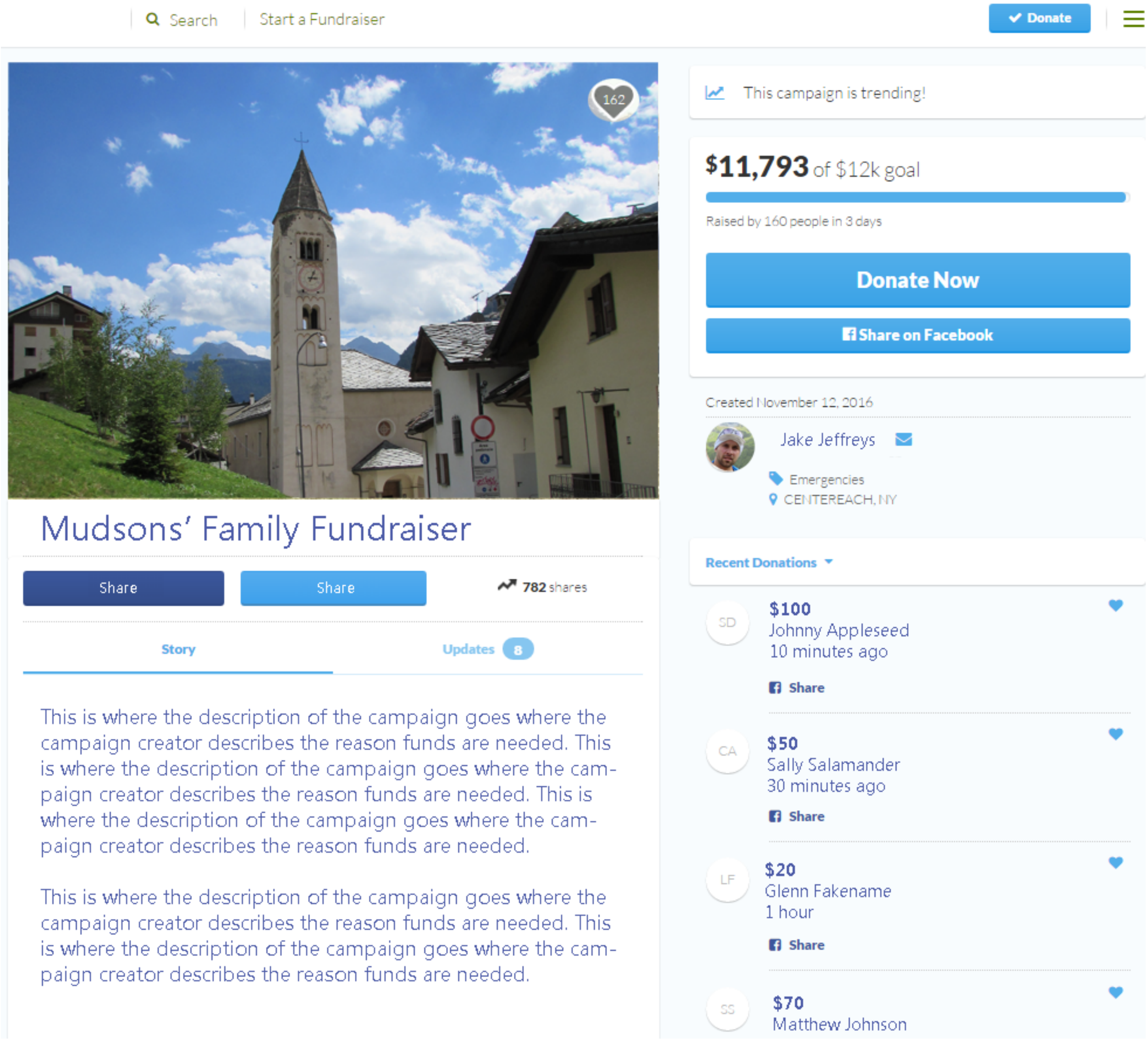

Supplementary Figure 1: Here you can see an anonymized screenshot of a GoFundMe page as users in our dataset saw them at the time of their donation decisions. In the bottom right you can see the listing of the most recent donations including the name and amount of the recent donors. If a donor left a message, it would also be displayed there.

2 Supplementary Methods

2.1 Gender estimation

2.1.1 Code

Below is the executable R code and some example output for our gender estimation procedure.

```
name_gender <- read.csv("C:/Users/Matt/Dropbox/go fund me/name_gender.csv",
                stringsAsFactors=FALSE)
head(name_gender)
```

```
##        name frequency cum_freq rank gender
## 1      MARY     2.629    2.629    1      F
## 2  PATRICIA     1.073    3.702    2      F
## 3     LINDA     1.035    4.736    3      F
## 4   BARBARA     0.980    5.716    4      F
## 5 ELIZABETH     0.937    6.653    5      F
## 6  JENNIFER     0.932    7.586    6      F
```

This table is from 1990 US Census. This dataset can be accessed here: http://www.census.gov/topics/population/genealogy/data/1990_census.html

Our gender estimation algorithm uses this table as follows:

```
estimate_gender <- function(name)
{
  if(grepl("&", name)){return(NA)}#get rid of people posting as a couple
  if(grepl(" and ", name)){return(NA)}
  if(grepl("family", name)){return(NA)}#or a whole family

  first_name <- strsplit(name, " ")[[1]][1]
  first_name <- toupper(first_name)
  sub <- subset(name_gender, name_gender$name==first_name)
  if (nrow(sub)==1) {return(sub$gender)}
  if (nrow(sub)==2) {
    sub <- sub[order(sub$frequency),]#order from least to greatest
    ratio <-sub$frequency[2] / sub$frequency[1]
    if (ratio > 10 ) {return(sub$gender[2])} else{return(NA)}#set the ratio
  }
  return(NA)
}
```

Here are some fictitious examples:

```
estimate_gender("John Smith")
```

```
## [1] "M"
```

```
estimate_gender("Mary Poppins")
```

```
## [1] "F"
```

```
estimate_gender("Sally & Tim Johnson")#should return NA as the name describes a couple
```

```
## [1] NA
```

Supplementary Table 1: A random sample of first names from our dataset that were estimated with a gender (not returned NA).

First name	Estimated gender
Dan	M
Brian	M
Bridget	F
Frances	F
Russell	M
Frances	F
Derek	M
Elena	F
Mark	M
Matthew	M
Gustavo	M
Ginny	F
Gwen	F
Theresa	F
Tom	M
Jason	M
Judy	F
Andrew	M
Dick	M
Josephine	F
Matt	M
Vanessa	F
Brian	M
Amanda	F
Cheryl	F
John	M
Lynne	F
Troy	M
Danielle	F
Andrew	M
Lianne	F
Stephanie	F
Laura	F
Laura	F
Bill	M
Ashley	F
Derek	M
Meghan	F
Cindy	F
LeeAnn	F

2.2 Descriptive statistics

2.2.1 Loading the data

Note: the file "Source_data_file_A.csv" is publicly available and linked to the main paper.

```
all_data <- read.csv("Source_data_file_A.csv", stringsAsFactors = F)

#Slight renaming for capitalization
library(dplyr)
all_data <- rename(all_data,
        Prop_visible_female = prop_visible_female,
        Same_last_name = same_last_name,
        Mean_visible_donation = mean_visible_donation)
```

The count of donations in our full dataset:

```
length(all_data$amount_donated)
```

```
## [1] 558067
```

The count of unique campaigns in our full dataset:

```
length(unique(all_data$campaign_ID))
```

```
## [1] 9264
```

The median of donations in our full dataset:

```
median(all_data$amount_donated)
```

```
## [1] 50
```

The mean and trimmed mean:

```
mean(all_data$amount_donated)#raw mean
```

```
## [1] 79.29079
```

```
mean(all_data$amount_donated[scale(all_data$amount_donated)<3])#mean with donations >=3SD removed
```

```
## [1] 71.18576
```

The sum of all donations (in US dollars):

```
sum(all_data$amount_donated)
```

```
## [1] 44249573
```

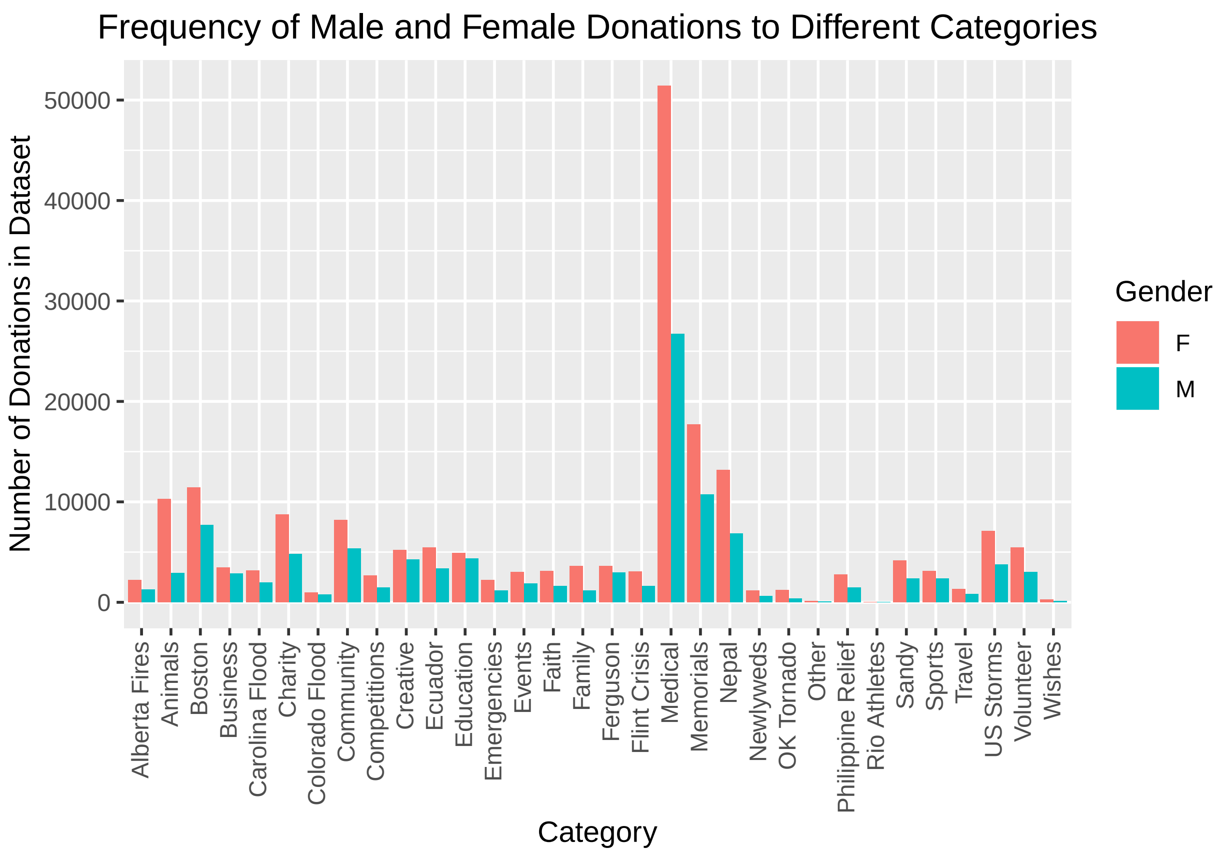

Supplementary Figure 2: Campaign categories by gender

2.2.2 Campaign categories by gender

Here you can see the frequency of male and female identified donations in our dataset to different campaign categories. The categories were provided on the GoFundMe website.

```
master_nhb <- subset(all_data, !is.na(all_data$gender))

bardata <- aggregate(amount_donated~gender+category, data=master_nhb, length)

bardata$Gender <- bardata$gender
bardata$Category <- bardata$category#to make uppercase in graph

require(ggplot2)
```

```
## Loading required package: ggplot2
```

```
ggplot(bardata, aes(fill=Gender, y=amount_donated, x=Category)) +
  ggtitle("Frequency of Male and Female Donations to Different Categories")+
  theme(plot.title = element_text(hjust = 0.5)) + ylab("Number of Donations in Dataset") +
  geom_bar(position="dodge", stat="identity") +
  theme(axis.text.x = element_text(angle = 90, hjust = 1, vjust=.4))
```

2.2.3 Anonymous donations

Below is the code and precise calculations for the statistics reported in the paper related to anonymous donations.

Obtaining the count of donations that were anonymous and not anonymous:

```
anon_table <- aggregate(amount_donated ~ anonymous, all_data, length)
anon_table
```

```
##   anonymous amount_donated
## 1         0         440193
## 2         1         117874
```

Calculating the proportion that were anonymous:

```
anon_table$amount_donated[2]/nrow(all_data)
```

```
## [1] 0.2112184
```

Calculating the standard error for the proportion:

```
SE <- sd(all_data$anonymous) / sqrt(nrow(all_data))
SE
```

```
## [1] 0.0005463884
```

Median anonymous donation:

```
median(all_data$amount_donated[all_data$anonymous==1])
```

```
## [1] 35
```

Sums of anonymous and non-anonymous donations:

```
anon_table <- aggregate(amount_donated ~ anonymous, all_data, sum)
anon_table
```

```
##   anonymous amount_donated
## 1         0       34002364
## 2         1       10247209
```

Proportion of dollars donated anonymously:

```
anon_table$amount_donated[2]/sum(anon_table$amount_donated)
```

```
## [1] 0.2315776
```

For this proportion we calculate the standard error through bootstrapping because donations are the units sampled, not individual dollars:

```
samples_anon_prop <- c()
for(i in 1:1e3)#number of bootstrap resamples set here
{
  resample <- all_data[sample(1:nrow(all_data),nrow(all_data), replace = T),]
  agg<-aggregate(amount_donated~anonymous, data=resample, sum)
  prop <- agg$amount_donated[2]/sum(agg$amount_donated)
  samples_anon_prop <- c(samples_anon_prop,prop)
}
```

The bootstrapped standard error:

```
sd(samples_anon_prop)
```

```
## [1] 0.003086065
```

2.3 Female and male donations

Below are the code snippets and output for the statistics reported in the paper related to male and female donations.

First subset the larger dataset to only those with donor gender estimated as male or female:

```
master_nhb <- subset(all_data, !is.na(all_data$gender))
```

Number of observations in subsetted dataset for gender analyses:

```
nrow(master_nhb)
```

```
## [1] 312613
```

Number of campaigns:

```
length(unique(master_nhb$campaign_ID))
```

```
## [1] 8987
```

Sum of donations:

```
sum(master_nhb$amount_donated)
```

```
## [1] 21543258
```

Count of donations by gender:

```
agg <- aggregate(amount_donated ~ gender, master_nhb, length)
agg
```

```
##   gender amount_donated
## 1      F         199473
## 2      M         113140
```

Proportion of donations by gender:

```
agg$proportion <- agg[,2]/sum(agg[,2])
agg
```

```
##   gender amount_donated proportion
## 1      F         199473  0.6380829
## 2      M         113140  0.3619171
```

Standard errors:

```
sd(master_nhb$gender=="F") / sqrt(nrow(master_nhb))
```

```
## [1] 0.0008594889
```

```
sd(master_nhb$gender=="M") / sqrt(nrow(master_nhb))
```

```
## [1] 0.0008594889
```

Null hypothesis (two-tailed) test with null: mu = .5

```
t.test(master_nhb$gender=="M", mu=.5)
```

```
##
##  One Sample t-test
##
## data:  master_nhb$gender == "M"
## t = -160.66, df = 312610, p-value < 2.2e-16
## alternative hypothesis: true mean is not equal to 0.5
## 95 percent confidence interval:
##  0.3602326 0.3636017
## sample estimates:
## mean of x
## 0.3619171
```

Effect size (Cohen's d):

```
(mean(master_nhb$gender=="M") - .5)/sd(master_nhb$gender=="M")
```

```
## [1] -0.2873399
```

Sum of donations by gender:

```
agg <- aggregate(amount_donated ~ gender, master_nhb, sum)
agg
```

```
##   gender amount_donated
## 1      F       11928532
## 2      M        9614726
```

Comparison of male vs. female mean donations:

```
t.test(amount_donated ~ gender, master_nhb, var.equal=FALSE)
```

```
##
##  Welch Two Sample t-test
##
## data:  amount_donated by gender
## t = -34.866, df = 145290, p-value < 2.2e-16
## alternative hypothesis: true difference in means is not equal to 0
## 95 percent confidence interval:
##  -26.59606 -23.76505
## sample estimates:
## mean in group F mean in group M
##        59.80023        84.98078
```

Effect size (Cohen's d):

```
male_mean <- mean(master_nhb$amount_donated[master_nhb$gender=="M"])
female_mean <- mean(master_nhb$amount_donated[master_nhb$gender=="F"])
```

```r
male_sd <- sd(master_nhb$amount_donated[master_nhb$gender=="M"])
female_sd <- sd(master_nhb$amount_donated[master_nhb$gender=="F"])

pooled_sd <- sqrt((male_sd^2 + female_sd^2)/2)

(male_mean - female_mean)/pooled_sd#Cohen's d
```

```
## [1] 0.1401921
```

Median of donations by gender:

```r
agg <- aggregate(amount_donated ~ gender, master_nhb, median)
agg
```

```
##   gender amount_donated
## 1      F             40
## 2      M             50
```

2.4 Regressions

Here we provide the code for running the regression reported in the paper, and the code and output for several regressions run as robustness checks and additional analyses.

2.4.1 Main regression from paper

```
#Dummy coding donor and recipient gender variables
table(master_nhb$gender)
```

```
##
##      F      M
## 199473 113140
```

```
master_nhb$Donor_gender_male <- ifelse(master_nhb$gender=="M", 1, 0)

table(master_nhb$recipient_gender)
```

```
##
##      F      M
## 150495  76979
```

```
master_nhb$Recipient_gender_male <- ifelse(master_nhb$recipient_gender=="M", 1, 0)
```

```
#Mean center the Mean_visible_donation variable
master_nhb$Mean_visible_donation <- scale(master_nhb$Mean_visible_donation, scale=F)

require(lme4)
lmer <- lmer(amount_donated ~  Donor_gender_male + Prop_visible_female
                  + Mean_visible_donation
                  + Same_last_name
                  + Donor_gender_male*Prop_visible_female
                  + Mean_visible_donation*Donor_gender_male
                  + Recipient_gender_male*Donor_gender_male
                  + (1|category) + (1|campaign_ID)
                  , master_nhb[abs(scale(master_nhb$amount_donated))<3 &
                    abs(scale(master_nhb$Mean_visible_donation))<3,]
             )
```

Supplementary Table 2: Regression results

	AMOUNT_DONATED
Donor_gender_male	13.889270***
	(.829195)
	t = 16.750310
	p = 0.000000
Prop_visible_female	−2.468032**
	(.784061)
	t = −3.147755
	p = .001646
Mean_visible_donation	.051933***
	(.003512)
	t = 14.788690
	p = 0.000000
Same_last_name	29.272200***
	(1.474831)
	t = 19.847830
	p = 0.000000
Recipient_gender_male	−1.151490
	(.796035)
	t = −1.446532
	p = .148029
Donor_gender_male:Prop_visible_female	3.290250**
	(1.150171)
	t = 2.860661
	p = .004228
Donor_gender_male:Mean_visible_donation	.068037***
	(.005034)
	t = 13.516220
	p = 0.000000
Donor_gender_male:Recipient_gender_male	−1.057022
	(.653141)
	t = −1.618366
	p = .105584
Constant	58.408380***
	(1.849873)
	t = 31.574260
	p = 0.000000
Observations	218,053
Notes:	*P < .05
	**P < .01
	***P < .001
	SE in parentheses

Supplementary Table 3: Calculating confidence intervals for the regression coefficients

	Estimate	Std. Error	lower	upper
(Intercept)	58.41	1.85	54.78	62.03
Donor_gender_male	13.89	0.83	12.26	15.51
Prop_visible_female	-2.47	0.78	-4.00	-0.93
Mean_visible_donation	0.05	0.00	0.05	0.06
Same_last_name	29.27	1.47	26.38	32.16
Recipient_gender_male	-1.15	0.80	-2.71	0.41
Donor_gender_male:Prop_visible_female	3.29	1.15	1.04	5.54
Donor_gender_male:Mean_visible_donation	0.07	0.01	0.06	0.08
Donor_gender_male:Recipient_gender_male	-1.06	0.65	-2.34	0.22

Calculating confidence intervals for the regression coefficients

```
CIs <- as.data.frame(coef(summary(lmer))[,1:2])

#construct 95% CIs
CIs$lower <- CIs[,1] - CIs[,2]*1.96
CIs$upper <- CIs[,1] + CIs[,2]*1.96
```

2.4.2 Regression with log(amount_donated)

As can be seen in the histogram of donation amounts (below), the distribution of donation amounts is right-skewed, even with outliers removed. As a non-normal distribution for the y variable can often lead to non-normal distributions of residuals, it can be suitable in this situation to log transform the y variable to make the distribution more normal.
Below you can see the effect of a log transformation on the amount_donated variable.

```
hist(master_nhb$amount_donated[scale(master_nhb$amount_donated)<3],100,
     main="Histogram of amount_donated")
```

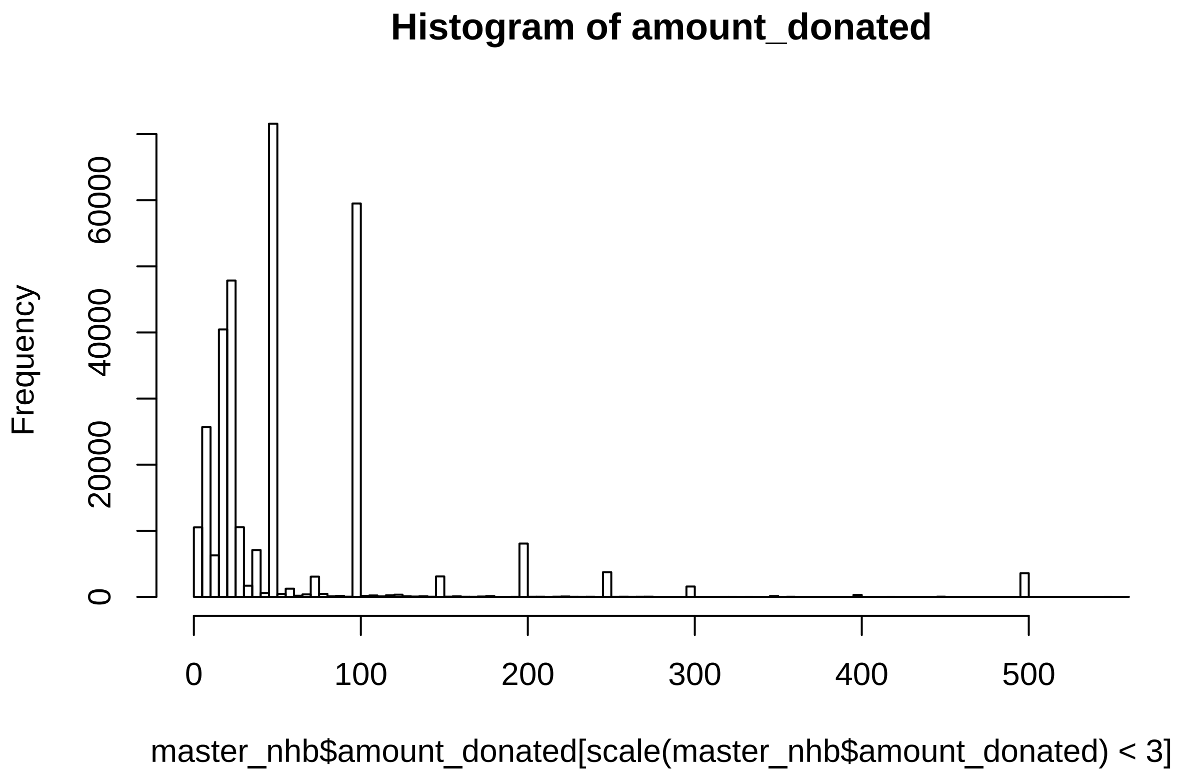

```r
hist(log(master_nhb$amount_donated[scale(master_nhb$amount_donated)<3]),100,
     main="Histogram of log(amount_donated)")
```

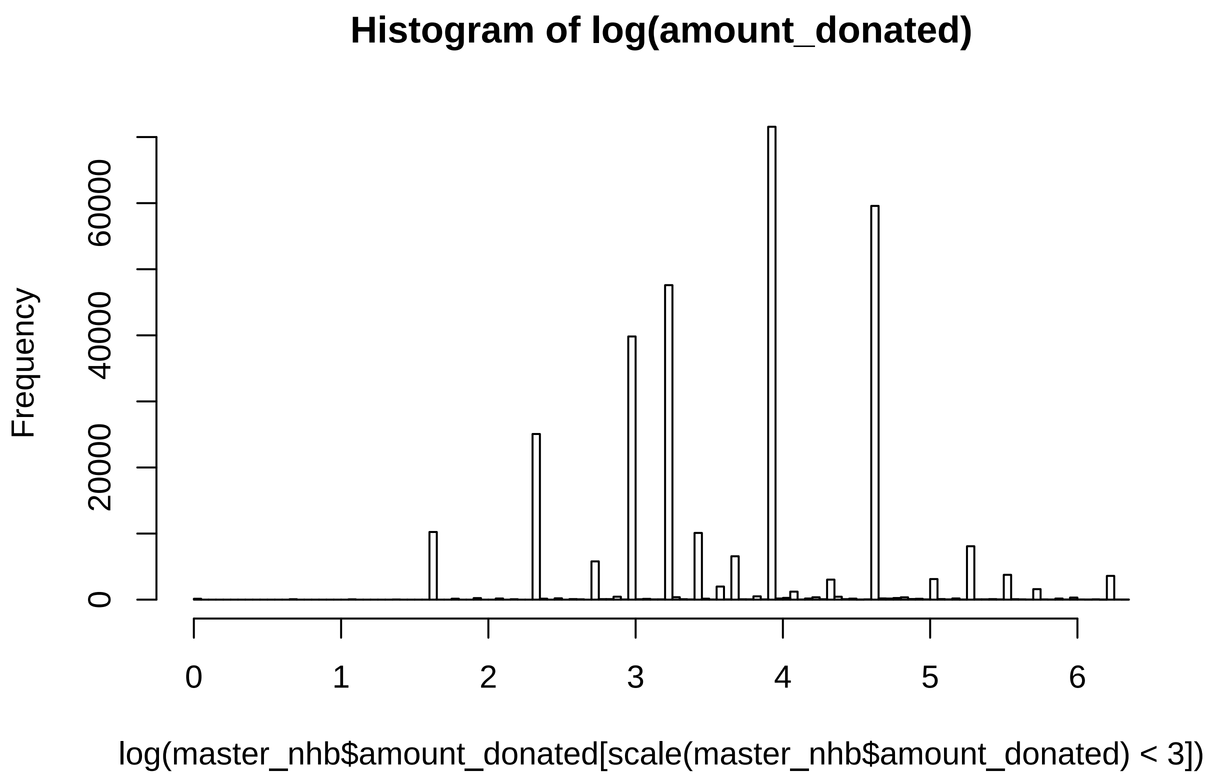

Indeed, the right-skew has been ameliorated. Technically, this should make for a better fit with the assumptions of our regression analysis. However, reporting our main regression table in log transformed donation amounts would make the interpretation throughout the paper much less comprehensible for readers. Thus, we show here that the patterns of effects with the log(amount_donated) and the raw amount_donated y variables are essentially the same. This justifies presenting and discussing the regression with the untransformed amount_donated variable in the paper.

```r
lmer_log <- lmer(log(amount_donated) ~  Donor_gender_male + Prop_visible_female
                  + Mean_visible_donation
                  + Same_last_name
                  + Donor_gender_male*Prop_visible_female
                  + Mean_visible_donation*Donor_gender_male
                  + Recipient_gender_male*Donor_gender_male
                  + (1|category) + (1|campaign_ID)
                  , master_nhb[abs(scale(master_nhb$amount_donated))<3 &
                    abs(scale(master_nhb$Mean_visible_donation))<3,]
            )
```

Supplementary Table 4: Regression results with raw vs log amount donated

	AMOUNT_DONATED	LOG(AMOUNT_DONATED)
	1	2
Donor_gender_male	13.889270***	.167140***
	(.829195)	(.010148)
Prop_visible_female	−2.468032**	−.023387*
	(.784061)	(.009695)
Mean_visible_donation	.051933***	.000780***
	(.003512)	(.000043)
Same_last_name	29.272200***	.337124***
	(1.474831)	(.018107)
Recipient_gender_male	−1.151490	−.027327*
	(.796035)	(.011958)
Donor_gender_male:Prop_visible_female	3.290250**	.027245
	(1.150171)	(.014089)
Donor_gender_male:Mean_visible_donation	.068037***	.000380***
	(.005034)	(.000062)
Donor_gender_male:Recipient_gender_male	−1.057022	−.029596***
	(.653141)	(.007993)
Constant	58.408380***	3.694189***
	(1.849873)	(.032337)
Observations	218,053	218,053
Notes:	*P < .05<br>**P < .01<br>***P < .001	

2.4.3 Regressions with recipient-oriented variables excluded

This regression below has a larger sample size than the one reported in the paper because it does not include recipient-oriented variables (e.g. recipient gender) which could not be coded for all recipients. We include this regression only in the supplementary materials as the results are almost identical to the regression in the main paper even though it has a smaller sample. One important difference is that in this regression the effect of *Prop_visible_female* is greater and more significant for male donors.

```
require(lme4)
lmer <- lmer(amount_donated ~  Donor_gender_male + Prop_visible_female
                  + Mean_visible_donation
                  # + Same_last_name
                  + Donor_gender_male*Prop_visible_female
                  + Mean_visible_donation*Donor_gender_male
                  + #Recipient_gender_male*Donor_gender_male
                  + (1|category) + (1|campaign_ID)
                  , master_nhb[abs(scale(master_nhb$amount_donated))<3 &
                    abs(scale(master_nhb$Mean_visible_donation))<3,]
            )
```

Supplementary Table 5: Regression results without recipient-oriented variables

	AMOUNT_DONATED
Donor_gender_male	12.885***
	(.654)
Prop_visible_female	−2.479***
	(.667)
Mean_visible_donation	.051***
	(.003)
Donor_gender_male:Prop_visible_female	4.083***
	(.975)
Donor_gender_male:Mean_visible_donation	.070***
	(.004)
Constant	58.320***
	(1.746)
Observations	292,669
Notes:	*P < .05
	**P < .01
	***P < .001

This next regression recodes the donor gender variable so that male=0 and female=1 (opposite of the coding used in the main regression in the paper). This allows us to test for the significance of the effect of *Prop_visible_female* for male donors. Since male now equals 0 the coefficient for *Prop_visible_female* is the effect of visible females on male donors. We see that it is statistically significant.

```
require(lme4)
master_nhb$Donor_gender_FEMALE <- ifelse(master_nhb$Donor_gender_male==1, 0, 1)
#reverse the coding
lmer <- lmer(amount_donated ~  Donor_gender_FEMALE + Prop_visible_female
                  + Mean_visible_donation
                  # + Same_last_name
                  + Donor_gender_FEMALE*Prop_visible_female
                  + Mean_visible_donation*Donor_gender_FEMALE
                  + #Recipient_gender_male*Donor_gender_FEMALE
                  + (1|category) + (1|campaign_ID)
                  , master_nhb[abs(scale(master_nhb$amount_donated))<3 &
                    abs(scale(master_nhb$Mean_visible_donation))<3,]
            )
```

Supplementary Table 6: Regression results without recipient-oriented variables - Donor gender recoded

	AMOUNT_DONATED
Donor_gender_FEMALE	−12.885***
	(.654)
Prop_visible_female	1.604*
	(.802)
Mean_visible_donation	.121***
	(.004)
Donor_gender_FEMALE:Prop_visible_female	−4.083***
	(.975)
Donor_gender_FEMALE:Mean_visible_donation	−.070***
	(.004)
Constant	71.205***
	(1.761)
Observations	292,669
Notes:	*P < .05
	**P < .01
	***P < .001

2.4.4 Regressions with various outlier cutoffs

In the main regression in the paper we use an outlier cutoff threshold of >=3 standard deviations from the average donation amount and the average visible donation. This is to remove the rare donors whom gave massive amounts, and the people who saw them on the page at the time of their donation decisions. As a robustness check, here we present the same regression run with two other outlier cutoff thresholds of >=2 SDs and >=1 SD. Also we include the results when no outliers are excluded. You can see that the results are essentially unchanged with lower cutoffs and are made more extreme but with the same pattern of significant findings when no outliers are removed.

```
require(lme4)
lmer1 <- lmer(amount_donated ~  Donor_gender_male + Prop_visible_female
                  + Mean_visible_donation
                  + Same_last_name
                  + Donor_gender_male*Prop_visible_female
                  + Mean_visible_donation*Donor_gender_male
                  + Recipient_gender_male*Donor_gender_male
                  + (1|category) + (1|campaign_ID)
                  , master_nhb[abs(scale(master_nhb$amount_donated))<1 & #keep where <1 SD
                    abs(scale(master_nhb$Mean_visible_donation))<1,]
            )

lmer2 <- lmer(amount_donated ~  Donor_gender_male + Prop_visible_female
                  + Mean_visible_donation
                  + Same_last_name
                  + Donor_gender_male*Prop_visible_female
                  + Mean_visible_donation*Donor_gender_male
                  + Recipient_gender_male*Donor_gender_male
                  + (1|category) + (1|campaign_ID)
                  , master_nhb[abs(scale(master_nhb$amount_donated))<2 &
                    abs(scale(master_nhb$Mean_visible_donation))<2,]
            )

lmer3 <- lmer(amount_donated ~  Donor_gender_male + Prop_visible_female
                  + Mean_visible_donation
                  + Same_last_name
                  + Donor_gender_male*Prop_visible_female
                  + Mean_visible_donation*Donor_gender_male
                  + Recipient_gender_male*Donor_gender_male
                  + (1|category) + (1|campaign_ID)
                  , master_nhb[abs(scale(master_nhb$amount_donated))<3 &
                    abs(scale(master_nhb$Mean_visible_donation))<3,]
            )

lmer4 <- lmer(amount_donated ~  Donor_gender_male + Prop_visible_female
                  + Mean_visible_donation
                  + Same_last_name
                  + Donor_gender_male*Prop_visible_female
                  + Mean_visible_donation*Donor_gender_male
                  + Recipient_gender_male*Donor_gender_male
                  + (1|category) + (1|campaign_ID)
                  , master_nhb#full dataset is used with no outliers removed
            )
```

Supplementary Table 7: Regression results at varying outlier cutoffs

	AMOUNT_DONATED			
	SD<1	SD<2	SD<3	all data
	1	2	3	4
Donor_gender_male	8.151***	9.720***	13.889***	21.789***
	(.493)	(.594)	(.829)	(2.055)
Prop_visible_female	−.707	−1.880***	−2.468**	−7.448***
	(.465)	(.563)	(.784)	(1.907)
Mean_visible_donation	.061***	.057***	.052***	.007**
	(.003)	(.003)	(.004)	(.002)
Same_last_name	15.786***	19.297***	29.272***	50.272***
	(.894)	(1.071)	(1.475)	(3.612)
Recipient_gender_male	−.698	−1.001	−1.151	−1.601
	(.503)	(.611)	(.796)	(1.522)
Donor_gender_male:Prop_visible_female	1.411*	2.931***	3.290**	7.016*
	(.684)	(.824)	(1.150)	(2.842)
Donor_gender_male:Mean_visible_donation	.035***	.049***	.068***	.036***
	(.004)	(.004)	(.005)	(.004)
Donor_gender_male:Recipient_gender_male	−1.571***	−1.137*	−1.057	.711
	(.388)	(.468)	(.653)	(1.618)
Constant	51.051***	54.892***	58.408***	64.696***
	(1.274)	(1.466)	(1.850)	(2.860)
Observations	207,600	214,499	218,053	220,176
Notes:	*P < .05<br>**P < .01<br>***P < .001<br>SE in parentheses			

2.4.5 Regression same name interactions

In the following regression we estimate coefficients in a model that includes interactions with Same_last_name and Mean_visible_donation as well as Same_last_name and Prop_visible_oppsex. (Prop_visible_oppsex is a transformation of Prop_visible_female that reverses the values for females so it always represents the proportion of visible donors on the page of the opposite sex of the current donor. This is performed to avoid the necessity of estimating and interpreting a three-way interaction with Same_last_name by Prop_visible_female by Donor_gender_male.) The results are exploratory but interesting as we note in the discussion section of the main paper.

```
master_nhb$Prop_visible_oppsex <- ifelse(master_nhb$Donor_gender_male==1,
                                         master_nhb$Prop_visible_female,
                                         (1-master_nhb$Prop_visible_female))

require(lme4)
lmer5 <- lmer(amount_donated ~  Donor_gender_male + Prop_visible_oppsex
                  + Mean_visible_donation
                  + Same_last_name
                  + Same_last_name*Mean_visible_donation
                  + Same_last_name*Prop_visible_oppsex
                  + Prop_visible_oppsex*Donor_gender_male
                  + Mean_visible_donation*Donor_gender_male
                  + Recipient_gender_male*Donor_gender_male
                  + (1|category) + (1|campaign_ID)
                  , master_nhb[abs(scale(master_nhb$amount_donated))<3 &
                    abs(scale(master_nhb$Mean_visible_donation))<3,]
            )
```

Supplementary Table 8: Regression with same name interactions

	AMOUNT_DONATED
Donor_gender_male	16.411***
	(.786)
Prop_visible_oppsex	2.262**
	(.786)
Mean_visible_donation	.051***
	(.004)
Same_last_name	21.048***
	(2.496)
Recipient_gender_male	−1.168
	(.796)
Mean_visible_donation:Same_last_name	.097***
	(.024)
Prop_visible_oppsex:Same_last_name	18.537***
	(4.420)
Donor_gender_male:Prop_visible_oppsex	−1.731
	(1.297)
Donor_gender_male:Mean_visible_donation	.067***
	(.005)
Donor_gender_male:Recipient_gender_male	−1.094
	(.653)
Constant	56.038***
	(1.781)
Observations	218,053
Notes:	*P < .05
	**P < .01
	***P < .001
	SE in parentheses

2.5 Empathy analysis

Counts of donations with messages:

```
table(master_nhb$gender[!is.na(master_nhb$message)])
```

```
##
##     F     M
## 84407 42232
```

2.5.1 Empathy detection algorithm

Below is the code used to classify each message as expressing empathy or not.

```
data$empathy <- 0
for( i in 1:nrow(data))
{
  if(length(grep("feel your", ignore.case=T, data$message[i]))>0){data$empathy[i] <- 1}
  if(length(grep("feel for", ignore.case=T, data$message[i]))>0){data$empathy[i] <- 1}
  if(length(grep("empath", ignore.case=T, data$message[i]))>0){data$empathy[i] <- 1}
  if(length(grep("pain", ignore.case=T, data$message[i]))>0){data$empathy[i] <- 1}
  if(length(grep("heartfelt", ignore.case=T, data$message[i]))>0){data$empathy[i] <- 1}
  if(length(grep("my heart", ignore.case=T, data$message[i]))>0){data$empathy[i] <- 1}
  if(length(grep("thoughts", ignore.case=T, data$message[i]))>0){data$empathy[i] <- 1}
  if(length(grep("thinking", ignore.case=T, data$message[i]))>0){data$empathy[i] <- 1}
}
```

2.5.2 Empathy estimation robustness evaluation

The code below implements a permutation-based robustness check. Simply put, it compares the sums of male and female messages expressing empathy using every possible combination of the full set of key phrases (e.g. "feel your") This demonstrates that the finding that females expressed more empathy than males is robust to any combination of the full set of phrases we used.

```
#Build table with binary variable for the presence of each keyword
empathy_matrix <- data.frame(grepl("feel your", ignore.case=T, master_nhb$message),
grepl("feel for", ignore.case=T, master_nhb$message),
grepl("empath", ignore.case=T, master_nhb$message),
grepl("pain", ignore.case=T, master_nhb$message),
grepl("heartfelt", ignore.case=T, master_nhb$message),
grepl("my heart", ignore.case=T, master_nhb$message),
grepl("thoughts", ignore.case=T, master_nhb$message),
grepl("thinking", ignore.case=T, master_nhb$message),
grepl("sorry", ignore.case=T, master_nhb$message)
)

cols <- ncol(empathy_matrix)
male_sums <- colSums(empathy_matrix[master_nhb$gender=="M",])
female_sums <- colSums(empathy_matrix[master_nhb$gender=="F",])

require(e1071)
require(gtools)
all_diffs <- data.frame()
```

```
iteration <- 1
for(i in 2:cols)
{
combos <- combinations(cols,i)

    for(x in 1:nrow(combos))
    {
    sub <- empathy_matrix[,combos[x,]]
    sub$empathy <- rowSums(sub)
    sub$empathy <- ifelse(sub$empathy>0, 1, 0)#limit at 1
    sub$gender <- master_nhb$gender
    sub$message <- master_nhb$message

    sub <- sub[sub$message!="" & !is.na(sub$message),]
    agg <- aggregate(empathy~gender, sub, mean)

    table <- table(sub$gender, sub$empathy)
    female_perc <- agg$empathy[1]
    male_perc <- agg$empathy[2]
    diff <- female_perc - male_perc
    results <- chisq.test(table)

    iteration <- iteration+1
    all_diffs <- rbind(all_diffs, c(diff, i, results$p.value))
    }
}
```

```
plot(1:nrow(all_diffs), all_diffs[,1], xlim=c(0,nrow(all_diffs)), ylim=c(-.01,.06),
     xlab="Combination", ylab="Percent More Empathy in Females",
     col=rainbow(cols)[all_diffs$X2], pch=all_diffs$X2)
title("Percent more female empathy in all combinations of key phrases")
abline(0,0, lty=3)
legend(450,.019, paste(c(2:cols), "Phrases"), col=rainbow(cols)[2:cols], pch=c(2:9), cex=.6)
```

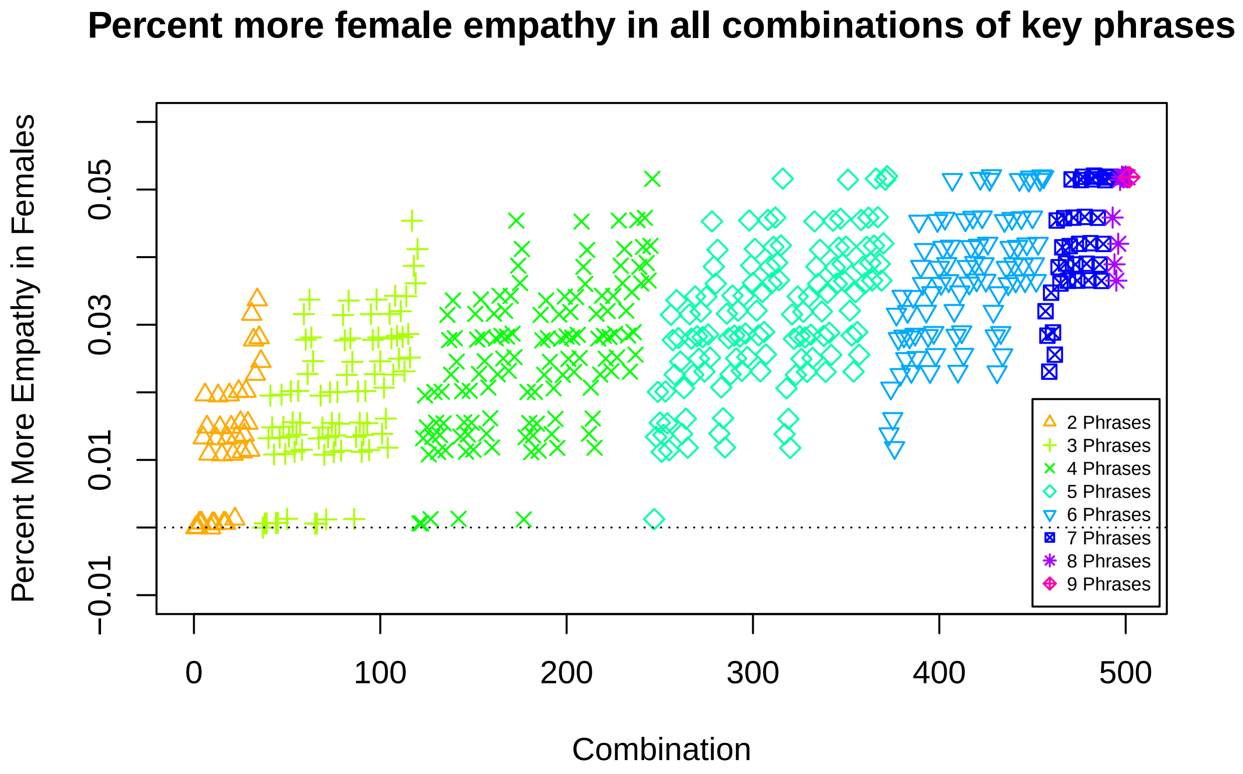

Proportion of combinations finidng women expressing more empathy:

```
mean(all_diffs>0)
```

```
## [1] 0.998008
```

2.5.3 Statistical analysis of empathy content

The percentage of male and female messages coded as expressing empathy:

```
agg <- aggregate(empathy ~ gender, master_nhb[!is.na(master_nhb$message),], mean)
agg
```

```
##   gender    empathy
## 1      F 0.12838983
## 2      M 0.08247301
```

Standard errors:

```
agg_n <- aggregate(empathy ~ gender, master_nhb[!is.na(master_nhb$message),], length)
agg_n
```

```
##   gender empathy
## 1      F   84407
## 2      M   42232
```

```
sqrt((agg[1,2]*(1-agg[1,2]))/agg_n[1,2])#females
```

```
## [1] 0.001151429
```

```
sqrt((agg[2,2]*(1-agg[2,2]))/agg_n[2,2])#males
```

```
## [1] 0.001338581
```

Chi-squared test of independence:

```
sub <- master_nhb[!is.na(master_nhb$message),]
table <- table(sub$gender, sub$empathy)
sum(table)
```

```
## [1] 126639
```

```
table
```

```
##
##         0     1
##   F 73570 10837
##   M 38749  3483
```

```
#chi squared test
chisq.test(table)
```

```
##
##  Pearson's Chi-squared test with Yates' continuity correction
##
## data:  table
## X-squared = 591.29, df = 1, p-value < 2.2e-16
```

2.6 Simulation: same name by chance

Here we demonstrate the probability of a donor and recipient having the same name by chance alone. We iteratively randomly match donors and recipients in our dataset and count the percentage of the matches we see in the original dataset that we would expect due to chance alone.

```
all_data_raw <- read.csv("all_data_raw.csv", stringsAsFactors = F)
sim_counts <- c()
for(i in 1:1e3)#repeat random matching 1000 times
{
  #randomly match donors to recipients and count how many times
  ##there is a same name match
  n_samename <- sum(tolower(all_data_raw$donor_lastname)==
    tolower(all_data_raw$creator_lastname[sample(1:nrow(all_data_raw))]), na.rm=T)
  n_samename
  sim_counts <- c(sim_counts, n_samename)
}

#how many same name matches occur by chance (averaged over 500 simulations)
mean(sim_counts)
```

```
## [1] 111.843
```

```
#calculate the number of same name matches in our original dataset
n_samename <- sum(tolower(all_data_raw$donor_lastname)==
                  tolower(all_data_raw$creator_lastname), na.rm=T)
n_samename
```

```
## [1] 5144
```

```
#what percentage of the same name matches in our dataset is this?
mean(sim_counts) / n_samename
```

```
## [1] 0.02174242
```

It would appear that having 2% of the same name matches in our data due to chance alone could not affect the final results very much. We quantify this precisely to ascertain the potential for bias due to 2% chance matches. We find below that for chance same name matches to entirely create the effect of $29.27 we see in our data, chance same name donations would need to be more than $1,300 on average.

```
#how much of an effect would the 2% chance matches need to have to alone create the results we find?
chance_effect <- seq(100,2000,100)
calc_mean <- function(chance_effect){mean(c(rep(chance_effect,mean(sim_counts)),
rep(0, n_samename-mean(sim_counts))))}
plot(chance_effect, sapply(chance_effect, FUN=calc_mean),
     ylab="Final estimate of same name effect",
     xlab="Effect of chance same name match",
     main="Translation of same name effect into final estimate")
abline(h=29.27, lty=3)

#calculate it exactly
necessary_effect <- (29.27*n_samename)/mean(sim_counts)
necessary_effect
```

```
## [1] 1346.216
```

```
abline(v=necessary_effect, lty=3)
```

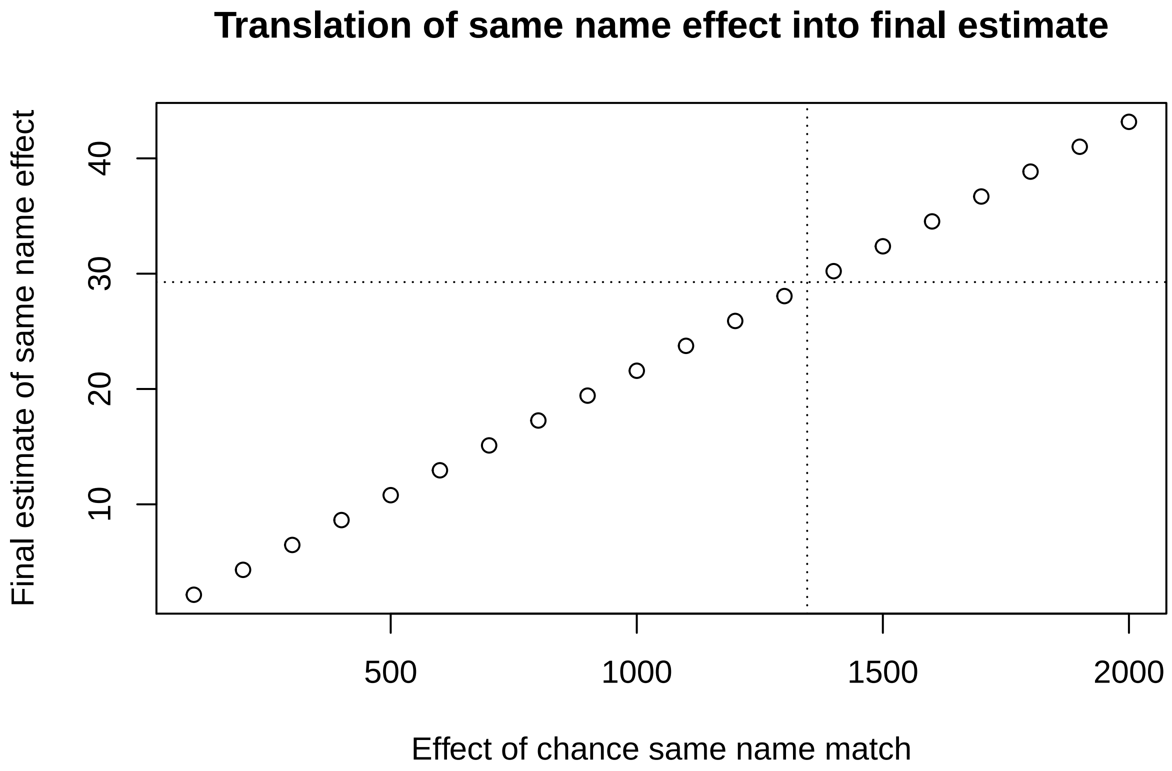

How much would a comparable effect of chance same name matches have on our estimate of a kin effect?

```
calc_mean(29.27)
```

```
## [1] 0.6317266
```

We estimate it would increase our estimate of the kin effect by about $0.63.

2.7 Simulation: controlling for campaign effects

Here we demonstrate the effectiveness of our method of controlling for campaign-level effects. Location or popularity of a campaign could make the donations for it on more or less compared to other campaigns. This is a sensible concern and our analysis effectively controls for this by estimating a random effect for each campaign as well as for each campaign category.

In this simulation, we created a dataset where we simulate donations to campaigns where previous donations do not have an influence on future ones, and we give campaigns different average donation amounts. We show that analyzing these data with a regression that does not control for campaign effects does skew the results, as one might expect. However this is not what we do in our analysis. When control for campaign-level effects (estimate random effects for campaigns) the bias disappears as we would expect it to.

```
#create a simulated dataset with six campaigns
sim_data <- data.frame(campaignid = c(rep("a",1000),rep("b",1000),rep("c",1000),rep("d",1000),
      rep("e",1000),rep("f",1000)),donation_number = c(rep(1:1000,6)))

head(sim_data)
```

```
##   campaignid donation_number
## 1          a               1
## 2          a               2
## 3          a               3
## 4          a               4
## 5          a               5
## 6          a               6
```

```
sim_data$amount_donated <- NA#create blank column for amount donated
sim_data$previous_average <- NA#store past 10

#first simulate with NO social influence effect
for(i in 1:nrow(sim_data))
{
    if(i%%1000 >= 11)
    {
    previous_average <- mean(sim_data$amount_donated[(i-11):(i-1)])
    #sim_data$amount_donated[i] <- rnorm(1, previous_average)
    sim_data$amount_donated[i] <- rnorm(1, 35)
    sim_data$previous_average[i] <- previous_average
    }else{
    sim_data$amount_donated[i] <- rnorm(1, 35)
    }
}

#visualizae the amount donated sequentially for each campaign
library(ggplot2)
ggplot(sim_data, aes(x=donation_number, y=amount_donated,
                     color=campaignid)) + geom_point() +
  facet_wrap(facets=vars(campaignid)) +
  ggtitle("Simulated campaign donations")
```

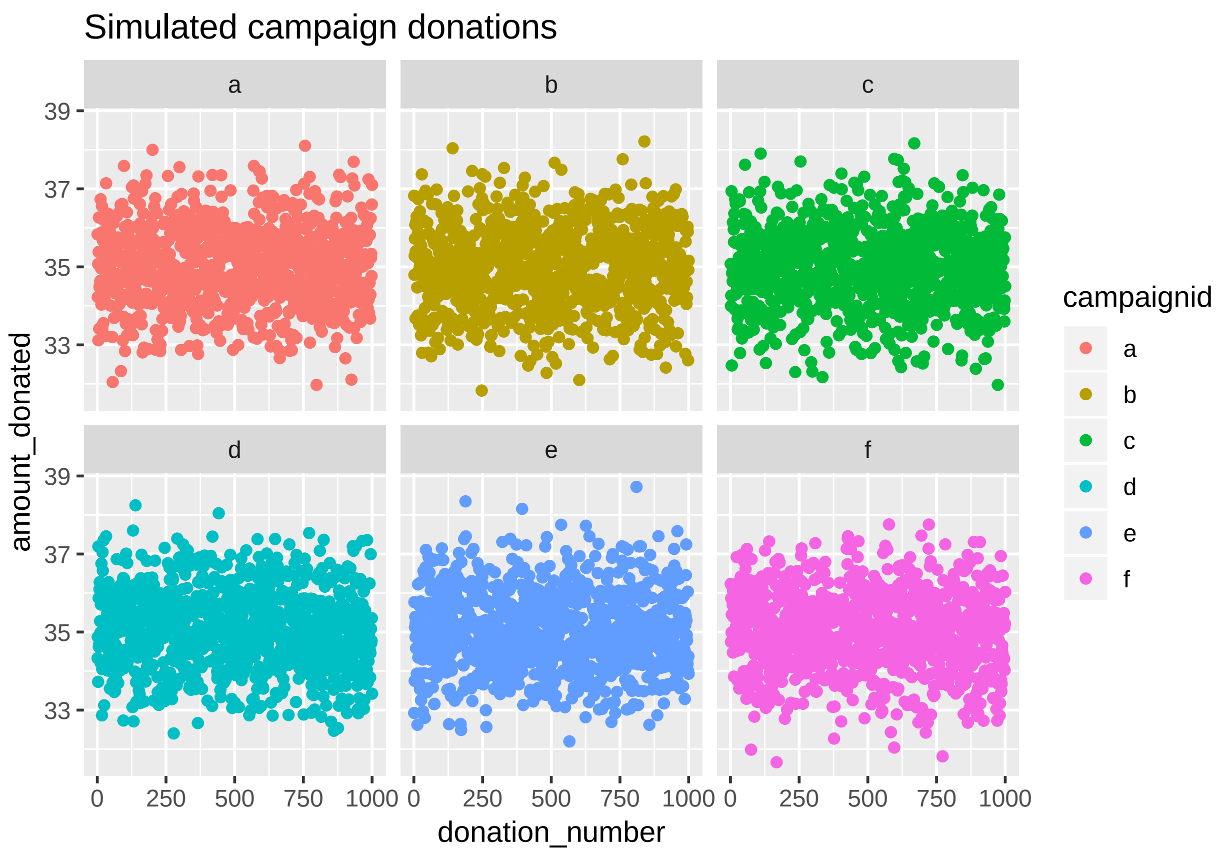


```
#now add an effect of campaigns
sim_data$amount_donated <- ifelse(sim_data$campaignid=="a",
                                  sim_data$amount_donated*2, sim_data$amount_donated)
sim_data$amount_donated <- ifelse(sim_data$campaignid=="b",
                                  sim_data$amount_donated+40, sim_data$amount_donated)
sim_data$amount_donated <- ifelse(sim_data$campaignid=="c",
                                  sim_data$amount_donated*.2, sim_data$amount_donated)
sim_data$amount_donated <- ifelse(sim_data$campaignid=="d",
                                  sim_data$amount_donated*1.2, sim_data$amount_donated)
sim_data$amount_donated <- ifelse(sim_data$campaignid=="e",
                                  sim_data$amount_donated+10, sim_data$amount_donated)
sim_data$amount_donated <- ifelse(sim_data$campaignid=="f",
                                  sim_data$amount_donated+25, sim_data$amount_donated)

ggplot(sim_data, aes(x=donation_number, y=amount_donated,
                     color=campaignid)) + geom_point() +
  facet_wrap(facets=vars(campaignid)) +
  ggtitle("Simulated campaign donations")
```

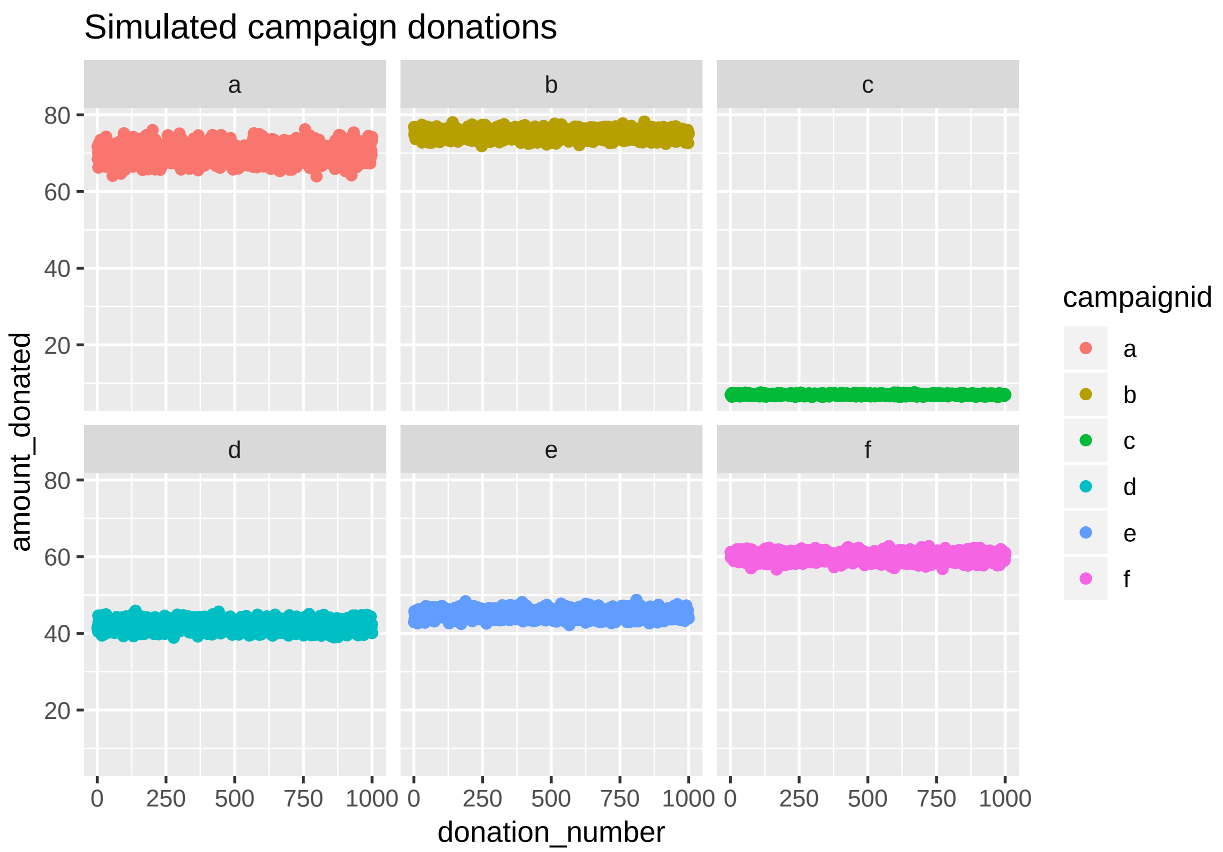


```
#without controlling for campaign effects we do see a marginal effect
#of previous donations which is spurious as they have not been
#added to the simulation yet
summary(lm(amount_donated ~ previous_average, sim_data))
```

```
##
## Call:
## lm(formula = amount_donated ~ previous_average, data = sim_data)
##
## Residuals:
##     Min      1Q  Median      3Q     Max
## -45.949  -7.530   2.439  20.080  30.084
##
## Coefficients:
##                  Estimate Std. Error t value Pr(>|t|)
## (Intercept)      168.9139    33.9509   4.975  6.7e-07 ***
## previous_average  -3.4020     0.9699  -3.507 0.000456 ***
## ---
## Signif. codes:  0 '***' 0.001 '**' 0.01 '*' 0.05 '.' 0.1 ' ' 1
##
## Residual standard error: 22.59 on 5932 degrees of freedom
##   (66 observations deleted due to missingness)
## Multiple R-squared:  0.00207,    Adjusted R-squared:  0.001901
## F-statistic:  12.3 on 1 and 5932 DF,  p-value: 0.0004558
```

```
#but when we control for campaign effects
library(lme4)
```

```r
summary(lmer(amount_donated ~ previous_average + (1|campaignid), sim_data))
```

```
## Linear mixed model fit by REML ['lmerMod']
## Formula: amount_donated ~ previous_average + (1 | campaignid)
##    Data: sim_data
##
## REML criterion at convergence: 19014.1
##
## Scaled residuals:
##     Min      1Q  Median      3Q     Max
## -5.0777 -0.5266  0.0033  0.5201  5.1975
##
## Random effects:
##  Groups     Name        Variance Std.Dev.
##  campaignid (Intercept) 611.804  24.735
##  Residual                 1.425   1.194
## Number of obs: 5934, groups:  campaignid, 6
##
## Fixed effects:
##                  Estimate Std. Error t value
## (Intercept)      48.87054   10.25828   4.764
## previous_average  0.02760    0.05162   0.535
##
## Correlation of Fixed Effects:
##             (Intr)
## previos_vrg -0.176
```

```r
#Now we correctly find no significant effect of past donations
```

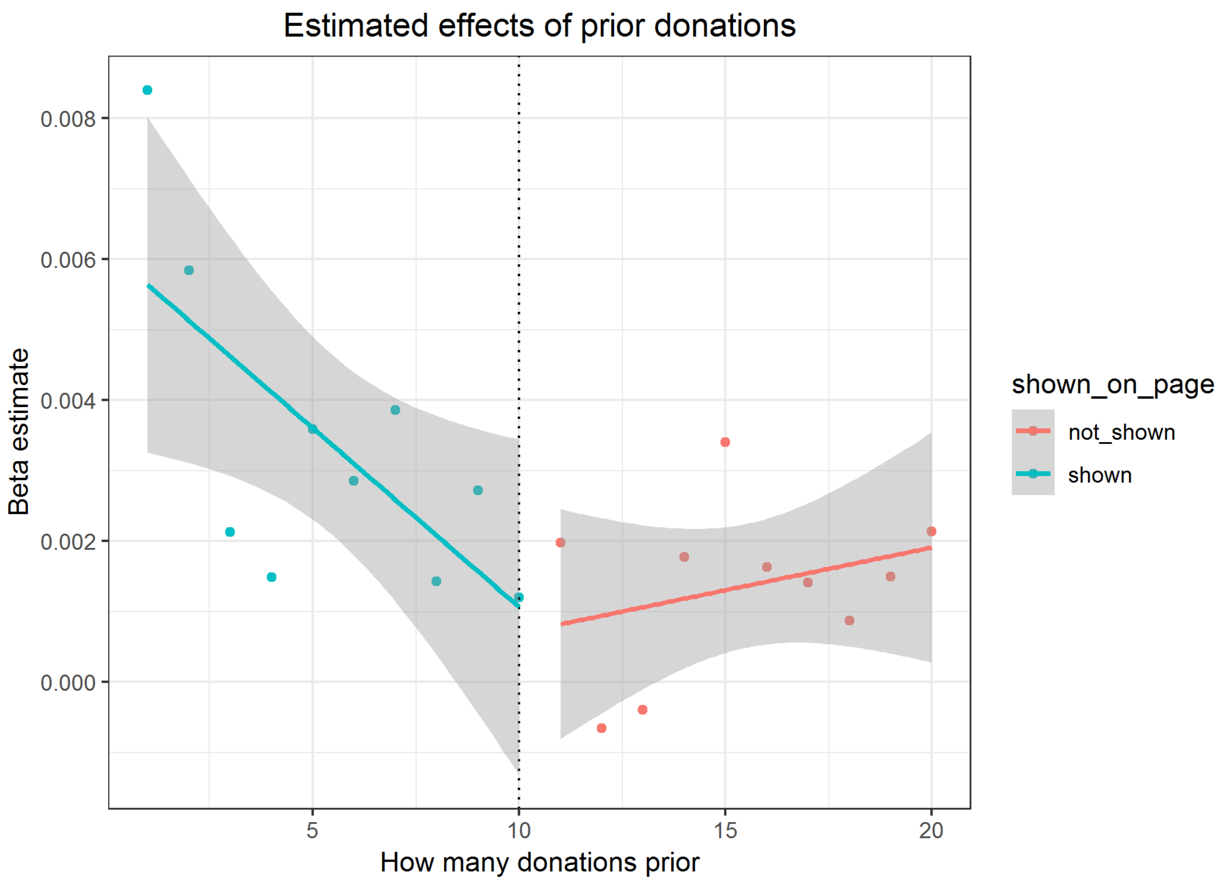

Supplementary Figure 3: Effects of prior individuals

2.8 Robustness check: investigating time as a confound

While campaign-level effects are controlled for in our analyses (as we just described), one reviewer's comment brought up the valid possibility of time as a confound within campaigns. For example, it could be that there are some days when people are just more generous in general, such as holidays. This could force some close-in-time donations to correlate with each other which could look like recent donations are influencing consecutive future ones.

Intuitively, it seems unlikely that generosity has enough systematic and widespread swings over time for this to substantially affect our results. Nonetheless, we investigated the possibility of this in the following way. We reason that if the effects of the past 10 donations (the donations on the screen at the time of each donation decision) are actually due to time confounds (or any other confound) then the influence of donations just after the threshold of 10 should be very similar.

If the correlation with past donations is truly due to their visibility, then we would expect to see a marked difference in the correlation between past donations up to the past ten and then a marked decline in correlation after that point.

You can see below that our findings are what we expected. There is an obvious difference between whether recent donations were shown on the screen or not (<=10 compared to >10 donations prior).

The visualization above shows the beta estimates for each prior donation from 1-20 donations prior. For robustness we repeated this analysis with ordered clusters of prior donations (1-3, 4-6, 7-9, etc) which you can see below.

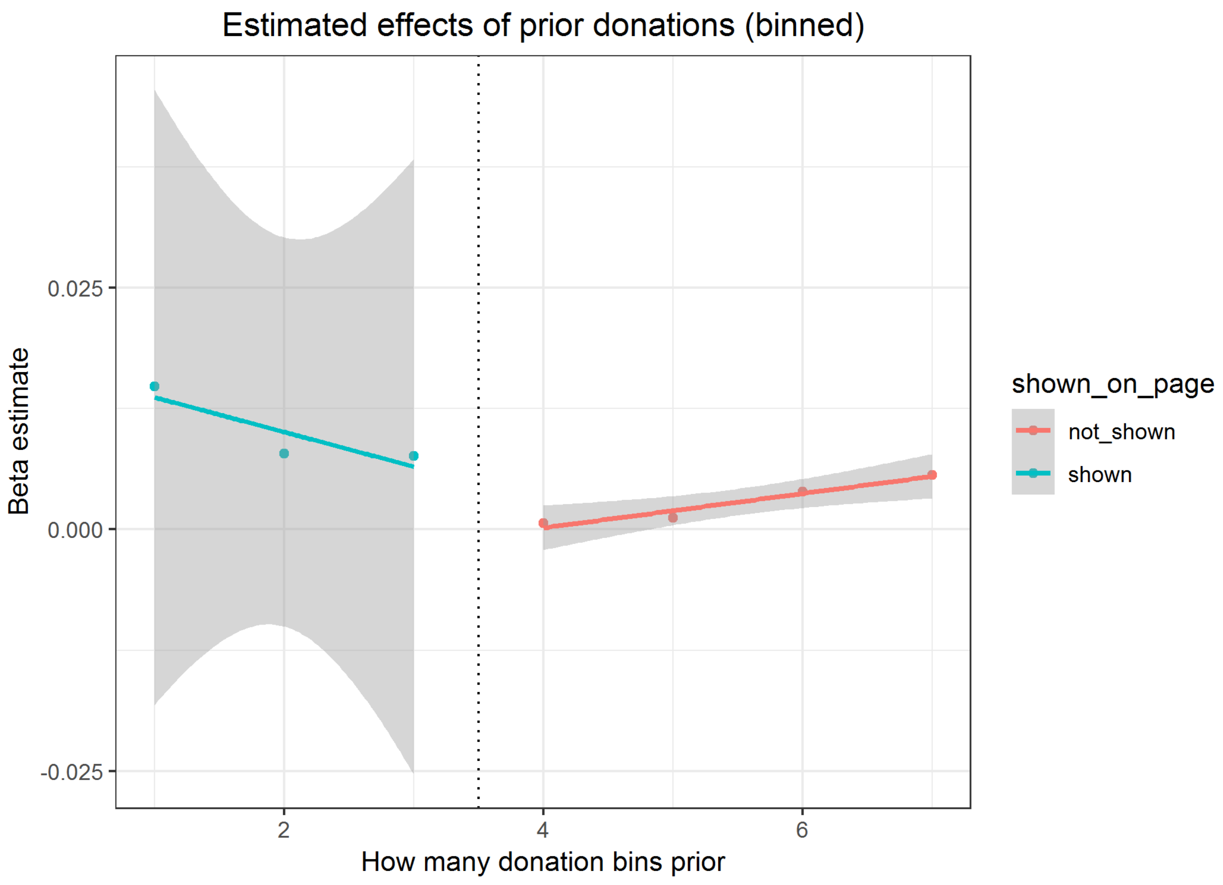

Supplementary Figure 4: Effects of prior donations - binned

These analyses support our claim that the effects of recent donations we find are due to the visibility of these donations and not attributable to a confound of time.

Supplementary Table 9: Minimum detectable effects

Variable	Estimate	Std__Error	t__value	minimum.detectable.effect
(Intercept)	58.41	1.85	31.57	3.63
Donor__gender__male	13.89	0.83	16.75	1.63
Prop__visible__female	-2.47	0.78	-3.15	1.54
Mean__visible__donation	0.05	0.00	14.79	0.01
Same__last__name	29.27	1.47	19.85	2.89
Recipient__gender__male	-1.15	0.80	-1.45	1.56
Donor__gender__male:Prop__visible__female	3.29	1.15	2.86	2.25
Donor__gender__male:Mean__visible__donation	0.07	0.01	13.52	0.01
Donor__gender__male:Recipient__gender__male	-1.06	0.65	-1.62	1.28

2.9 Sensitivity (Post hoc) Power Analysis

Here we show the minimum detectable effect (SE * 1.96; alpha=5%) for each of the coefficients in our main regression.

2.10 Survey data analysis

2.10.1 Code and analysis to produce Figure 1:

```
data <- read.csv("Source_data_file_B.csv", stringsAsFactors = F)
first_col <- 6#where the motivation columns start
motivation_means <- as.data.frame(colMeans(data[,first_col:ncol(data)]))
colnames(motivation_means) <- c("proportion")
motivation_means$motivation <- row.names(motivation_means)


library(binom)#calculate confidence intervals (95%)
motivation_means <- cbind(motivation_means,
                    binom.exact(motivation_means$proportion*nrow(data),nrow(data)))

clean_labels <- function(x){#clean the column labels for graphing
  x <- gsub("_", " ", x)
  substr(x, 1, 1) <- toupper(substr(x, 1, 1))
  x
  }

motivation_means <- motivation_means[order(motivation_means$proportion),]#reorder
motivation_means$motivation <- factor(motivation_means$motivation,
  levels=motivation_means$motivation[order(motivation_means$proportion)])
  #make factor variable to preserve order

library(ggplot2)#code for producing Figure 1 in paper
p <- ggplot(motivation_means, aes(x=motivation, y=proportion, fill=motivation)) +
  geom_bar(stat="identity") +  coord_flip()+ theme_bw()+ theme(legend.position="none")+
  xlab("Motivations for donating")  + ylab("Proportion of donors") +
  ggtitle("Primary motivation for donating") +
  theme(plot.title = element_text(hjust = 0.5), text = element_text(size=25))+
  geom_errorbar(aes(ymin=lower, ymax=upper),
  width=.2)+
  scale_x_discrete(labels=
  clean_labels(motivation_means$motivation[order(motivation_means$proportion)]))
```

```r
library(knitr); kable(motivation_means, digits=2, row.names=F,
      caption="Proportions of Primary Motivations")
```

Supplementary Table 10: Proportions of Primary Motivations

proportion	motivation	method	x	n	mean	lower	upper
0.00	future_reciprocity	exact	1	305	0.00	0.00	0.02
0.00	save_on_taxes	exact	1	305	0.00	0.00	0.02
0.00	to_attract_mates	exact	1	305	0.00	0.00	0.02
0.01	donor_directly_benefitted	exact	4	305	0.01	0.00	0.03
0.02	to_avoid_guilt	exact	6	305	0.02	0.01	0.04
0.03	recipient_was_relative	exact	10	305	0.03	0.02	0.06
0.04	other	exact	11	305	0.04	0.02	0.06
0.08	feel_like_better_person	exact	24	305	0.08	0.05	0.11
0.09	felt_empathy	exact	28	305	0.09	0.06	0.13
0.16	helping_feels_good	exact	49	305	0.16	0.12	0.21
0.53	recipient_needed_help	exact	163	305	0.53	0.48	0.59

2.10.2 Code and analysis of anonymous donors' responses

How many anonymous donor respondents reported being motivated by absolutely none of the egoistic ones:

```
#the nonegoistic column equals = 1 if they reported "Yes"
#to none of the egoistic motivation responses.
sum(data$Donations.Anony=="Yes")
```

```
## [1] 173
```

```
mean(data$nonegoistic[data$Donations.Anony=="Yes"])
```

```
## [1] 0.1098266
```

```
altruistic <- mean(data$nonegoistic[data$Donations.Anony=="Yes"])

SE <- sqrt((altruistic)*(1-altruistic)/sum(data$Donations.Anony=="Yes"))
SE
```

```
## [1] 0.02377213
```

```
#t test; null: true proportion=0
#also calculates confidence interval
t.test(data$nonegoistic[data$Donations.Anony=="Yes"], mu=0)
```

```
##
##  One Sample t-test
##
## data:  data$nonegoistic[data$Donations.Anony == "Yes"]
## t = 4.6066, df = 172, p-value = 7.938e-06
## alternative hypothesis: true mean is not equal to 0
## 95 percent confidence interval:
##  0.06276772 0.15688546
## sample estimates:
## mean of x
## 0.1098266
```

```
#cohen's d:
altruistic / sd(data$nonegoistic[data$Donations.Anony=="Yes"])
```

```
## [1] 0.3502334
```

2.11 Survey instrument

Below you can find the full survey instrument for the supplemental survey.

The finding reported in the manuscript that 11% of anonymous donors reported no egoistic motivations at all is based on questions below. The questions highlighted in light blue are the ones that participants must have answered negatively to all of in order to be considered reporting no egoistic motivations.

Several questions in the survey instrument were not analyzed for this paper, but we include the questions anyway to convey the survey exactly as it was taken by participants.

GoFundMe Donors Supplementary Survey

Screening Question Have you ever used the website, GoFundMe, to make a donation?

- ○ Yes (1)
- ○ No (2)

Display This Question:

If Have you ever used the website, GoFundMe, to make a donation? = No

Sorry, if you did not donate on GoFundMe you cannot take this study (as we said in the HIT description). **Please return the hit.**

Please complete the captcha.

[Informed Consent occurred here]

How many times have you donated on the GoFundMe site?

- ○ 1-5 times (1)
- ○ 6-10 times (2)
- ○ more than 10 times (3)

What was the amount of the **most recent donation** you made on GoFundMe?

- ○ $1-$15 (1)
- ○ $16-$30 (3)
- ○ $31-$50 (4)
- ○ $51-$75 (5)
- ○ $76-$100 (6)
- ○ $101 or more (7)

Thinking about your **most recent donation** on GoFundMe, can you describe in a few sentences why you made this donation?

____

____

____

____

____

Can you think of any ways that you benefited or could benefit from making this donation? Please describe.

____

____

____

____

____

Again, thinking about your most recent donation on GoFundMe:
Please select any and all of the reasons below **that motivated you** to donate.
Please take your time and be as honest as possible.

- ▢ I felt bad about something, and donating could help me feel better (1)
- ▢ I wanted to avoid feeling guilty for not helping (12)
- ▢ Helping someone makes me feel good (2)
- ▢ Helping someone makes me feel like a better person (11)
- ▢ The person I donated to needed the help (3)
- ▢ The person I donated to was a relative (4)
- ▢ Helping someone now increases the chances someone will help me later (5)
- ▢ Donating helps me save money on my taxes (6)
- ▢ If the campaign reached its goal it would directly benefit me (7)
- ▢ I could feel what the person in need was feeling (8)
- ▢ Being seen as a generous person makes me more attractive as a romantic partner (9)
- ▢ Other: (10) ____

Please put the options you selected in the order that they influenced your decision to donate. 1 being “influenced it the most”. Drag your answers to change the order.
______ I felt bad about something, and donating could help me feel better (1)
______ I wanted to avoid feeling guilty for not helping (2)
______ Helping someone makes me feel good (3)
______ Helping someone makes me feel like a better person (4)
______ The person I donated to needed the help (5)
______ The person I donated to was a relative (6)
______ Helping someone now increases the chances someone will help me later (7)
______ Donating helps me save money on my taxes (8)
______ If the campaign reached its goal it would directly benefit me (9)
______ I could feel what the person in need was feeling (10)
______ Being seen as a generous person makes me more attractive as a romantic partner (11)
______ Other: (12)

Imagine that a relative and a non-relative have identical campaign pages requesting contributions, and you want to contribute to both. How much more or less **would you give to a relative** in comparison to a non-relative?

○ A lot more (1)

○ Somewhat more (2)

○ The same amount (3)

○ Somewhat less (4)

○ A lot less (5)

Thinking about all donations made on GoFundMe by all contributors, what percentage of them do you think are made anonymously (meaning with names not publicly displayed with their donation)?

____

Are you a robot completing this survey automatically?

- Yes (1)
- No (2)

Were any of your past donations on GoFundMe made anonymously (meaning without your name being publicly displayed)?

- Yes (1)
- No (2)

Skip To: Largest anondonation If Were any of your past donations on GoFundMe made anonymously (meaning without your name being pub... = Yes

Skip To: Q28 If Were any of your past donations on GoFundMe made anonymously (meaning without your name being pub... = No

Thinking about your **most recent anonymous** donation on GoFundMe:

Please select any and all of the reasons below **that motivated you** to make this anonymous donation.
Please take your time and be as honest as possible.

- ▢ I felt bad about something, and donating could help me feel better (1)
- ▢ I wanted to avoid feeling guilty for not helping (12)
- ▢ Helping someone makes me feel good (2)
- ▢ Helping someone makes me feel like a better person (11)
- ▢ The person I donated to needed the help (3)
- ▢ The person I donated to was a relative (4)
- ▢ Helping someone now increases the chances someone will help me later (5)
- ▢ Donating helps me save money on my taxes (6)
- ▢ If the campaign reached its goal it would directly benefit me (7)
- ▢ I could feel what the person in need was feeling (8)
- ▢ Being seen as a generous person makes me more attractive as a romantic partner (9)
- ▢ Other: (10) ____

Again, thinking about your **most recent <u>anonymous</u>** donation on GoFundMe:

Why did you choose to make the donation **anonymously** instead of publicly?

____

____

____

____

____

Do you remember ever telling someone about this anonymous donation?

- ◯ Yes (1)
- ◯ No (2)

Did you include this anonymous donation in your taxes to receive a reduction?

- ◯ Yes (1)
- ◯ No (2)

Was the donation to a relative or non-relative?

- ◯ Relative (1)
- ◯ Non-relative (2)

How did you feel after making this donation?

- ○ Better than before (1)
- ○ About the same (2)
- ○ Worse than before (3)

Did you directly benefit in any way from making this donation?

- ○ Yes (1)
- ○ No (2)

Had the thought ever crossed your mind to donate anonymously in order to eventually mention to someone that you donated anonymously and impress them with the fact that you donated anonymously?

- ○ Yes (1)
- ○ No (2)

Lastly, just a few demographic questions.

gender What is your gender?

- ○ Male (1)
- ○ Female (2)
- ○ Other (3)

year_born What year were you born?

____

income What currently is your personal annual income before taxes? (Round to the nearest thousand)

____

Thanks for completing our survey! Your completion code is: ${rand://int/10000:99999}
