# Supplementary material for: Examining charitable giving in real-world online donations
Source: Nat Commun. 2019 Sep 3;10:3968. doi: 10.1038/s41467-019-11852-z (PMC6722054; doi:10.1038/s41467-019-11852-z)
Supplement: Supplementary file 3 — Reporting Summary [file 41467_2019_11852_MOESM3_ESM.pdf]

nature research

Corresponding author(s): Matthew R. Sisco

Reporting Summary

Nature Research wishes to improve the reproducibility of the work that we publish. This form provides structure for consistency and transparency in reporting. For further information on Nature Research policies, see Authors & Referees and the Editorial Policy Checklist.

Statistical parameters

When statistical analyses are reported, confirm that the following items are present in the relevant location (e.g. figure legend, table legend, main text, or Methods section).

n/a	Confirmed	
☐	☒	The exact sample size (n) for each experimental group/condition, given as a discrete number and unit of measurement
☐	☒	An indication of whether measurements were taken from distinct samples or whether the same sample was measured repeatedly
☐	☒	The statistical test(s) used AND whether they are one- or two-sided<br>*Only common tests should be described solely by name; describe more complex techniques in the Methods section.*
☐	☒	A description of all covariates tested
☐	☒	A description of any assumptions or corrections, such as tests of normality and adjustment for multiple comparisons
☐	☒	A full description of the statistics including central tendency (e.g. means) or other basic estimates (e.g. regression coefficient) AND variation (e.g. standard deviation) or associated estimates of uncertainty (e.g. confidence intervals)
☐	☒	For null hypothesis testing, the test statistic (e.g. F, t, r) with confidence intervals, effect sizes, degrees of freedom and P value noted<br>*Give P values as exact values whenever suitable.*
☒	☐	For Bayesian analysis, information on the choice of priors and Markov chain Monte Carlo settings
☒	☐	For hierarchical and complex designs, identification of the appropriate level for tests and full reporting of outcomes
☐	☒	Estimates of effect sizes (e.g. Cohen's d, Pearson's r), indicating how they were calculated
☐	☒	Clearly defined error bars<br>*State explicitly what error bars represent (e.g. SD, SE, CI)*

Our web collection on statistics for biologists may be useful.

Software and code

Policy information about availability of computer code

Data collection	The data were collected using the open source statistical software R (version 3.3).
Data analysis	The data were analyzed using the open source statistical software R (version 3.5). All R code necessary to reproduce the analyses is included in the supplementary materials, with relevant R output also displayed there.

For manuscripts utilizing custom algorithms or software that are central to the research but not yet described in published literature, software must be made available to editors/reviewers upon request. We strongly encourage code deposition in a community repository (e.g. GitHub). See the Nature Research guidelines for submitting code & software for further information.

Data

Policy information about availability of data

All manuscripts must include a data availability statement. This statement should provide the following information, where applicable:

- Accession codes, unique identifiers, or web links for publicly available datasets
- A list of figures that have associated raw data
- A description of any restrictions on data availability

The data that support the findings of this study will be made publicly available after the paper is published via a public repository. All of the code needed to replicate the analyses presented with these data is included in the Supplementary Information.

Field-specific reporting

Please select the best fit for your research. If you are not sure, read the appropriate sections before making your selection.

☐ Life sciences ☒ Behavioural & social sciences ☐ Ecological, evolutionary & environmental sciences

For a reference copy of the document with all sections, see nature.com/authors/policies/ReportingSummary-flat.pdf

Behavioural & social sciences study design

All studies must disclose on these points even when the disclosure is negative.

Study description	The primary data are observational and quantitative publicly accessible records of charitable donations. We also analyze supplemental survey data.
Research sample	The donation data: the donors are mostly US residents (presumably, as all donations analyzed are in US dollars). The only demographic information known for the donors is their genders (which is estimated from their first names). Out of the donations for which we could identify the gender of the donor, 64% were made by females.<br><br>The survey data: we recruited 331 participants through Amazon Mechanical Turk and prior to analysis removed 16 for failing an attention check. We reviewed the open ended responses to identify inappropriate responses and removed ten participants for leaving unacceptable (automated or incomprehensible) responses or ones that indicated they donated on different crowdfunding platform than GoFundMe. This left a sample of 305 responses. Our final sample was 47% male and had a mean age of 33 (SD=11). The modal amount donated was $16-$30.
Sampling strategy	We sought to download all donations that were available on the GoFundMe website at the time of data retrieval. We first collected a list of campaign identifiers from the GoFundMe website, then we iterated through the list of campaigns to download all publicly displayed donations to each campaign. The survey participants were recruited through Amazon Mechancial Turk. We spaced out out postings of the survey over several days to boost the diversity of the sample. Only participants who had previously donated on GoFundMe were permitted to take the survey.
Data collection	The records of donations on GoFundMe were downloaded from the GoFundMe.com website in May and June of 2016 by downloading and parsing the publicly accessible HTML. For the gender estimation algorithm we used publicly available data from the US Census, available here: http://www.census.gov/topics/population/genealogy/data/1990_census.html. The survey data were collected using the survey software Qualtrics.
Timing	Data were downloaded during May and June of 2016. The donations actually took place between January 2012 and June 2016. The survey data were collected in February and March 2019.
Data exclusions	Different subsets of the full donation dataset were used for different analyses. Which subset is used for each analysis is described clearly in the body of the paper. To briefly summarize here, the full dataset is analyzed for the proportions of anonymous donations. Then we gender estimated the names of donors and only further analyzed observations where the name of the donor could be estimated. Lastly, for the regression reported in the paper, we include a variable for the gender of the recipient and therefore any donations made to recipients whose names we could not gender estimate are also not included. In the supplementary materials we show the regression results when all recipients are included. In the regression analysis we also exclude donations above three standard deviations from the mean, and donations that were made with a donation three or more standard deviations from the mean displayed on the page. In the supplementary materials we also show the regression results when no outliers are excluded, and at different cutoff thresholds.<br><br>For the survey data, as explained in the paper, prior to analysis we removed 16 responses for failing an attention check. We reviewed the open ended responses to identify inappropriate responses and removed ten participants for leaving unacceptable (automated or incomprehensible) responses or ones that indicated they donated on different crowdfunding platform than GoFundMe. This left a sample of 305 responses for the main analyses.
Non-participation	All participants that began the study completed it.
Randomization	These are not experimental studies. We did not allocate donors to different groups in the donation data. Some natural experiments are capitalized on in the study, namely what donors saw at the time of the donation decisions (i.e. proportion of females, average donation). This is treated as pseudo-random in our analyses and interpretations. We do control for the gender of the donors and recipients in the regression analysis.<br><br>There was also no randomization involved in the survey data as it was not an experiment.

Reporting for specific materials, systems and methods

Materials & experimental systems

n/a	Involved in the study
☒	☐ Unique biological materials
☒	☐ Antibodies
☒	☐ Eukaryotic cell lines
☒	☐ Palaeontology
☒	☐ Animals and other organisms
☐	☒ Human research participants

Methods

n/a	Involved in the study
☒	☐ ChIP-seq
☒	☐ Flow cytometry
☒	☐ MRI-based neuroimaging

Human research participants

Policy information about studies involving human research participants

Population characteristics	See above
Recruitment	Potential participants saw the survey under the title, "Five minute survey - *for GoFundMe users only*". If they clicked into it they then saw the description:<br><br>"Five-minute survey FOR USERS OF GoFundMe ONLY<br>ONLY accept this HIT if you have made a donation on GoFundMe in the past. Thank you!"<br><br>Participants were then asked again in the survey if they have donated in the past on GoFundMe and were shown the GoFundMe logo to aid their memories.
